# Effects of vitamin D and/or magnesium supplementation on mood, serum levels of BDNF, inflammatory biomarkers, and SIRT1 in obese women: a study protocol for a double-blind, randomized, placebo-controlled trial

**DOI:** 10.1186/s13063-020-4122-9

**Published:** 2020-02-26

**Authors:** Behnaz Abiri, Mohammadreza Vafa

**Affiliations:** 1 Department of Nutrition, Faculty of Paramedicine, Ahvaz Jundishapur University of Medical Sciences, Ahvaz, Iran; 2 Department of Nutrition, School of Public Health, Iran University of Medical Sciences, Tehran, Iran

**Keywords:** Vitamin D, Magnesium, Mood, Brain-derived neurotrophic factor, Inflammatory biomarkers, Sirtuin 1, Obese women

## Abstract

**Background:**

Emerging evidence has shown that vitamin D and magnesium have anti-inflammatory and anti-depressant effects. Dietary intake of magnesium is associated with reduced body mass index, waist circumference, body fat percentage, as well as inflammatory biomarkers and depressive symptoms. Vitamin D deficiency has been linked to inflammation, obesity, and depressive symptoms. This study will test the effects of vitamin D and magnesium co-supplementation on mood, serum level of brain-derived neurotrophic factor (BDNF), inflammation, and sirtuin 1 (SIRT1) in obese women.

**Methods:**

We will conduct an 8-week, double-blind, randomized, placebo-controlled clinical trial, in a factorial design, to evaluate the individual effects of vitamin D and magnesium, and co-supplementation of them, on mood, serum level of BDNF, inflammation, and SIRT1 in 108 obese women.

**Discussion:**

We hypothesize that vitamin D and magnesium co-supplementation may provide a new adjuvant therapy through modulation of BDNF, inflammation, and SIRT1 in obese women.

**Trial registration:**

Iranian Registry of Clinical Trials, IRCT20090822002365N23. Registered on 16 August 2019.

## Background

Depression is a global problem with widespread public health concern [1]. It affects 300 million people globally with a lifetime prevalence of 17% and has been projected to be the largest contributor to disease burden by the year 2030 (https://www.who.int/mental_health/management/depression/wfmh_paper_depression_wmhd_2012.pdf). Depression is linked with high morbidity and mortality, and can have a large negative socioeconomic impact due to associated functional disabilities and knock-on healthcare expenditures [2, 3]. Treatment options consist of antidepressant medications and/or psychotherapy, which are effective in relieving some of the effects of depression. However, over half of all people on these medications experience side effects, which can result in discontinuation of treatment [4]. Therefore, there is an urgent need for new treatment approaches with high efficacy and low side-effect profiles.

In a similar manner, obesity is also a global public health problem. In 2016, approximately 1.9 billion adults were classified as overweight (body mass index (BMI) > 25 kg/m^2^), with one-third of these being obese (BMI > 30 kg/m^2^) (https://www.who.int/news-room/fact-sheets/detail/obesity-and-overweight). As with depression, obesity is associated with decreased quality of life and reduced life expectancy [5]. This is due to the fact that obesity is also a major risk factor for non-communicable diseases such as heart disease, stroke, diabetes, and some cancers [6]. Recent evidence has emerged that there is a bidirectional relationship between obesity and depression as the occurrence of one of these disorders increases the risk of developing the other [7]. Thus, further studies are needed to increase our understanding of the underlying mechanisms associated with both conditions, which may lead to improved treatment options.

Obesity has been found to increase the risk of comorbid depression by 55%, and depression leads to a 58% increased risk of developing obesity [8]. Also, a number of epidemiological and meta-studies have demonstrated that obesity and depression are frequent co-occurring medical conditions [9–14]. Investigations on the mechanisms linking these two disorders have come to the consensus of dysfunctions in neuroendocrine, inflammation, and oxidative stress networks [15, 16]. A number of studies have now shown that depression is marked by chronic inflammation and that treatment with anti-cytokine therapies ameliorated the depressive symptoms [8, 17]. Likewise, the metabolic disruptions in obesity can result in systemic inflammation and treatments with anti-inflammatory agents are considered a novel therapeutic approach to ameliorate the obesity-related insulin resistance and other metabolic disturbances [18].

A number of other studies have also demonstrated that vitamin and mineral deficiency may play a role in both of these disorders. Magnesium is a vital element in higher organisms which regulates biochemical processes in multiple organ systems, including the brain [19]. A preclinical study showed that mice with low magnesium levels had increased belligerent behavior, with elevated levels of noradrenalin compared to control mice with normal magnesium levels [20]. Clinical studies found an inverse relationship between circulating magnesium concentrations and depressive symptoms [21–24]. Furthermore, one study showed that consumption of 500 mg magnesium oxide tablets for 8 weeks by depressed patients with magnesium deficiency led to improvements in depressive symptoms and magnesium levels [25]. Other studies have found antidepressant-like activities of magnesium supplementation in bipolar disorder [26], in women with premenstrual syndrome [27], in individuals with chronic fatigue syndrome [28], and in elderly individuals with depression, hypomagnesemia, and type 2 diabetes [29].

Magnesium deficiency can result in changes in cellular function, leading to the onset of metabolic disorders associated with inflammation, particularly in obese individuals with hypomagnesemia [30–32]. Some studies reported that dietary intake of magnesium is inversely related to physiometric measures such as BMI, waist circumference, and body fat composition [33, 34]. Hypomagnesemia also leads to decreased antioxidant enzyme activities, with concomitant increased production of damaging reactive oxygen species (ROS) by inflammatory cells [35].

Several studies have shown that vitamin D is essential for normal brain development and function, and vitamin D deficiency has been linked with neurological disorders, including depression [36–38]. Vitamin D is also involved in the initial biosynthetic stages of serotonin, a neurotransmitter which has been implicated in both depression and the mechanism of action of antidepressant drugs [39]. Vitamin D deficiencies have also been linked to obesity [38]. For example, studies have found inverse relationships of vitamin D with total body fat and metabolic syndrome [40]. A study also found decreased hippocampal levels of brain-derived neurotrophic factor (BDNF) in a preclinical model of depression, and these were normalized by vitamin D administration [38].

The well-known link of BDNF with depression has led to its frequent use as a potential biomarker in psychiatric disorders [41, 42]. However, its link with obesity is not well understood. In a rodent study, BDNF was found to regulate appetite via modulation of melanocortin signaling [38]. Furthermore, vitamin D deficiency has been associated with serum concentrations of inflammatory biomarkers including interleukin (IL)-6, tumor necrosis factor (TNF)-α, and C-reactive protein (CRP) in obese individuals [43], and increased dietary intake of vitamin D has been linked with lower visceral adiposity and adipocyte size [43].

Other metabolic processes have also been linked with both obesity and depression. The sirtuins are NAD^+^-dependent protein deacetylases in most cells with known roles in metabolism, the stress response, and aging [44]. Levels of SIRT1 have been found to be low in obesity and aging, and normalization of SIRT1 levels decreases disease effects [44]. Studies have also found that SIRT1 promotes insulin signaling through an anti-inflammatory mechanism and suppresses proinflammatory gene expression in adipocytes [45]. Also, the reverse association between SIRT1 and adipose tissue mass and inflammation was found to be normalized by vitamin D [43].

On the other hand, multiple steps in vitamin D metabolism and function require magnesium as a cofactor, including vitamin D binding to vitamin D binding protein, 25(OH)D synthesis, 1,25(OH)_2_D synthesis, 25-hydroxylase synthesis, and vitamin D receptor expression. In addition, serum 1,25(OH)_2_D levels remain low in individuals with magnesium deficiency even following vitamin D intake [46]. Magnesium deficiency has also been found to reduce parathyroid hormone production and the number of vitamin D receptors in target cells [46].

There is not enough information about the individual effect of vitamin D and magnesium on mood, BDNF, and SIRT1 and about co-supplementation. The aim of this study is to evaluate the effects of vitamin D and magnesium co-supplementation on behavioral measures of mood, circulating BDNF and inflammation-related biomarkers, and levels of SIRT1 in obese women.

## Methods

### Design

**Fig. 1:**
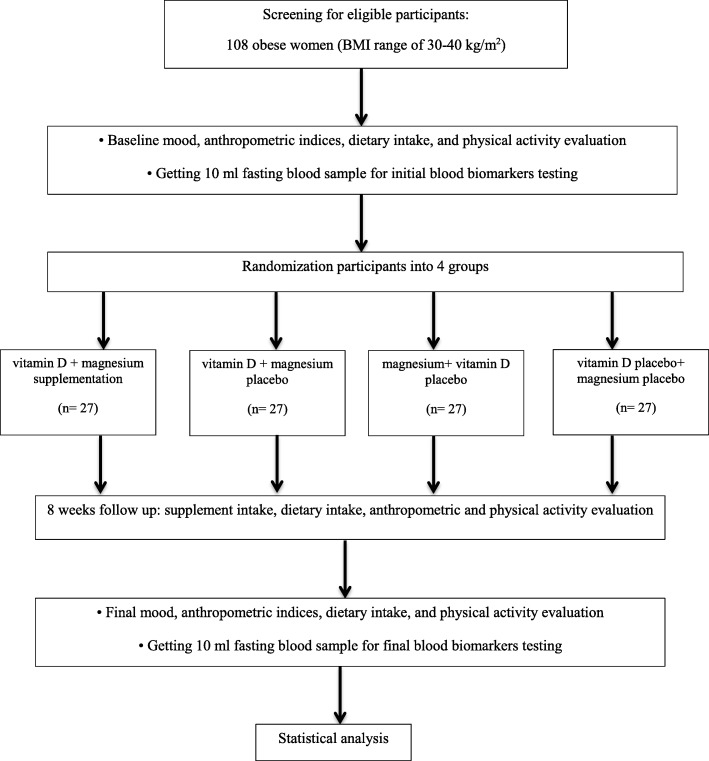
Protocol flow diagram; we will carry out an 8-week, double-blind, randomized controlled trial to determine the effects of vitamin D and/or magnesium supplementation on mood, serum level of brain-derived neurotrophic factor (BDNF), inflammation, and sirtuin 1 (SIRT1) in obese women. BMI body mass index

**Fig. 2:**
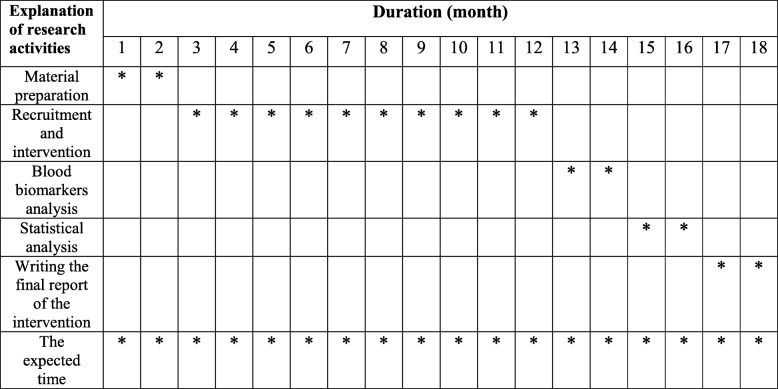
Timeline of the study; we expect 18 months for this trial

We will conduct an 8-week, double-blind, randomized controlled trial in a factorial design. Participants will be recruited from staff at the Iran University of Medical Sciences through advertisements. The trial will be conducted over 8 weeks to evaluate the effects of vitamin D_3_ (50,000 IU, one dose weekly) and magnesium (250 mg magnesium oxide, one dose daily) in obese women. The output measures will be behavioral readouts of mood, circulating levels of BDNF, cytokines, and SIRT1 (Figs. 1 and 2). The protocol is written in line with the Standard Protocol Items: Recommendations for Interventional Trials (SPIRIT) checklist (Additional file 1).

### Objectives and hypotheses of the study

The primary objective of the present study is to assess the effects of vitamin D_3_ and magnesium co-supplementation in obese women on readouts of mood, serum levels of 25(OH)-D, magnesium, BDNF, inflammatory biomarkers (hs-CRP, TNF-α, IL-6), and sirtuin 1 levels. For controlling of confounder factors, the study will evaluate anthropometric indices (weight, body mass index, and waist circumference), dietary intake of energy and macronutrients, and physical activity levels in the women at baseline and at the end of the study.

### Participants

Participants will be 108 obese women. The inclusion criteria will consist of: BMI = 30–40 kg/m^2^; age = 18–50 years; serum 25(OH)D < 30 ng/ml; serum magnesium ≤ 2 mg/dl; no indication of hormonal changes, autoimmune disease, diabetes, hypertension, or renal, hepatic, parathyroid, hormonal, and gastrointestinal disorders; not pregnant, breast-feeding, or menopaused; not taking vitamin D and/or magnesium supplements and other vitamin–mineral supplements or antidepressant drugs; not taking laxative and/or anti-inflammatory medications; and not having had a weight loss of ≥ 3 kg within 2 months leading up to the trial. Exclusion criteria will be less than 90% compliancy with the treatment.

### Ethics and trial registration

The patients who meet the inclusion criteria will be completely informed about the study protocol. The protocol of this study was approved by the Medical Ethics Committee of National Institute for Medical Research Development (NIMAD), and is in conformity with the Declaration of Helsinki (approval number: IR.NIMAD.REC.1398.181). Each participant will sign the informed consent form. This clinical trial was registered on Iranian Registry of Clinical Trials (IRCT registration number: IRCT20090822002365N23) which is available online (http://irct.ir/user/trial/20288/view).

### Sample size

To estimate the sample size, we considered α = 0.05 and β = 0.20 in line with previous trials. In addition, we used 1570.5 ng/ml as the standard deviation and 1250.0 ng/ml as the mean of hs-CRP levels as the primary variable, as described previously [47]. This resulted in a requirement of 25 participants in each group. To allow for attrition, 27 participants will be recruited for each group, giving a total of 108 obese women.

### Randomization

Randomization to the different treatment arms will be performed using computer-generated random numbers. The 108 participants who meet the criteria will be allocated as such into four treatment groups: 50,000 IU vitamin D soft gel (weekly), plus a 250-mg magnesium tablet (daily); 50,000 IU vitamin D soft gel (weekly) plus a magnesium placebo (daily); vitamin D placebo (weekly) plus a 250-mg magnesium tablet (daily); and a vitamin D placebo (weekly) plus a magnesium placebo (daily). The vitamin D soft gels and magnesium tablets are provided by Zahravi, Iran and Jalinous, Iran, respectively. The placebos for vitamin D and magnesium contained oral paraffin and maltodextrin, respectively, and are supplied by Zahravi, Iran (these were matched in appearance of the corresponding soft gels and tablets). Patients will receive verbal and written counseling on how to consume the supplements and compliance will be evaluated by tablet count every 2 weeks.

For blinding, a person who is not involved in the study protocol will create a randomized list assigning participants to each treatment arm. The soft gels, tablets, and corresponding placebos will be placed into unlabeled identical containers. The study leader will label the containers with participant numbers using the codes generated through the randomization. All investigators and participants will be blinded.

Women who meet the inclusion criteria will be informed completely about the protocol of the study. The ethics committee will review and approve the final study protocol. Each participant will sign the informed consent form.

### Outcome measures

Mood will be assessed using the validated Iranian version of the Beck Depression Inventory (BDI) [48] at baseline and after the intervention period. Blood samples will be collected at both time points after a 12-h overnight fast. Serum will be prepared according to standard protocols and stored at –80 °C until analyses. Serum 25(OH)D will be measured by enzyme-linked immune sorbent assay (ELISA) using the Euro Immun kit (Euro Immun, Germany). Serum magnesium will be determined using a spectrophotometric method with an autoanalyzer (Hitachi 912; Roche Diagnostics). Serum BDNF will be assessed by ELISA using the ZellBio kit (ZellBio, Germany). Serum hs-CRP values will be measured using an immunoturbidimetric method (Pars Azmun, Iran). Serum TNF-α and IL-6 levels will be assessed by ELISA using the Bender Med kit (Bender Med, Germany). Sirtuin 1 will be evaluated using ELISA with the ZellBio kit (ZellBio, Germany).

A questionnaire regarding patient medications and probable supplement use will be recorded at the beginning of the intervention. Dietary intake will be evaluated by 3 days of 24-h recall questionnaires (2 week days and 1 weekend day) at baseline and at the end of the intervention, and total energy and macronutrient intake will be estimated using Nutritionist ӀV software. Physical activity levels will be assessed using the short form of the International Physical Activity Questionnaire (IPAQ) at baseline and at the end of the intervention. Physiometric measures will be taken after overnight fasting, with minimal clothing and without shoes, at baseline and after the intervention. Body weight will be measured with an accuracy of 0.1 kg using the Beurer scale (Beurer, Germany). Height will be assessed using a stadiometer to the nearest 0.5 cm in a standing position without shoes. BMI will be computed as body weight (kilograms) divided by height squared (centimeters squared). Waist circumference will be evaluated at the narrowest point above the hips with an unstretched tape measure.

### Statistical analysis

Statistical tests of the outcome measures will be performed using SPSS (version 22.0; SPSS Inc., Chicago, IL, USA). The normality of distribution will be examined and confirmed by the Kolmogorov–Smirnov test. All results will be expressed as mean ± SD. Categorical variables will be presented as frequencies and percentages. The chi-square test will be used to test for differences between categorical variables. Baseline mean differences will be tested using one-way analysis of variance (ANOVA). Analysis of covariance (ANCOVA) will be used to identify differences between the four treatment arms at the end of study, adjusting for baseline values and covariates. The comparison of mean values will be done within groups after the intervention period using paired-sample *t* tests. Bonferroni corrections will be used in pairwise comparisons of the values after the intervention. *P* < 0.05 will be considered statistically significant.

## Discussion

Mounting evidence has demonstrated a two-way relationship between obesity and depression as a commonly co-occurring medical situation. Furthermore, both diseases have shown significant links with inflammation. Moreover, metabolic disruptions in obesity can result in increased levels of cortisol, leptin, and insulin, leading to dysregulation of neuroendocrine networks and insulin resistance. In turn, these conditions can lead to further inflammatory effects and worsen depression.

Moreover, the literature has demonstrated that vitamin and mineral deficiency can play a critical role in the pathogenesis of multiple disorders such as obesity and depression with clinical repercussions. Magnesium is one of the most vital elements in the human body and an obvious relationship between circulating magnesium concentrations and depressive symptoms has been shown in some studies. Furthermore, magnesium deficiency is a nutritional problem that results in alterations in cellular function and biological activity of the resident molecules, which may lead to the onset of metabolic disorders associated with inflammatory process. This situation is particularly pronounced in obese individuals with low serum and dietary levels of magnesium.

Additionally, several studies have reported that vitamin D deficiency is related to both depressive symptoms and obesity. Studies of obesity have also demonstrated effects on major central nervous system biomarkers, such as a decrease in BDNF levels in the hippocampus with reversal of the effect following vitamin D administration. SIRT1 is widely located in mammalian cells. Expression and enzymatic activity of SIRT1 are low in many non-communicable diseases, and SIRT1 activation can delay or ameliorate many of these effects. In addition, SIRT1 protects against diet-induced obesity and inflammation as well as obesity-related metabolic dysfunction.

Given the known role of vitamin D and magnesium deficiency in the pathogenesis of many non-communicable disorders, we will test the effects of their co-supplementation in obese women on the readouts of mood and serum levels of BDNF, inflammatory cytokines, and SIRT1.

## Trial status

The present research is at the stage of preparing the material. Protocol version number 2, dated 18 October 2019. Recruitment began on 20 October 2019, and will be completed on 20 December 2020.

## Supplementary information

**Additional file 1.** SPIRIT 2013 Checklist: Recommended items to address in a clinical trial protocol and related documents.

## Data Availability

Not applicable.
